# Experiences and Expectations of Hospitalised Patients Undergoing Negative Pressure Wound Therapy With Instillation: A Qualitative Study

**DOI:** 10.1111/scs.70203

**Published:** 2026-02-11

**Authors:** Annabel Nanninga, Heleen Westland, Ricardo G. Orsini, Marja A. Boermeester, Anne M. Eskes, Hannah Groenen

**Affiliations:** ^1^ Nursing Sciences, Program in Clinical Health Sciences University Medical Center Utrecht, Utrecht University Utrecht the Netherlands; ^2^ Department of Imaging and Oncology University Medical Center Utrecht Utrecht the Netherlands; ^3^ Julius Center for Health Sciences and Primary Care University Medical Center Utrecht Utrecht the Netherlands; ^4^ Department of Surgery Maastricht University Medical Center+ Maastricht the Netherlands; ^5^ Department of Surgery Amsterdam UMC Location University of Amsterdam Amsterdam the Netherlands; ^6^ Amsterdam Gastroenterology Endocrinology & Metabolism Amsterdam the Netherlands; ^7^ Amsterdam Public Health Amsterdam the Netherlands

**Keywords:** negative pressure wound therapy with instillation, patient experiences, qualitative study, semi‐structured interview, treatment impact, wound healing

## Abstract

**Aims and Objectives:**

Negative pressure wound therapy with instillation is increasingly used to treat various wounds. However, there is limited information about its impact on patient experiences. This study aims to explore the experiences of hospitalised patients undergoing negative pressure wound therapy with instillation for different types of wounds and to explore their expectations regarding continuing treatment at home.

**Methodological Design and Justification:**

We conducted a qualitative study using semi‐structured interviews. 12 patients treated with negative pressure wound therapy with instillation across various hospitals in the Netherlands were interviewed between January 2024 and June 2024. Data were analysed using Braun and Clarke's reflexive thematic analysis.

**Ethical Issues and Approval:**

This study was deemed not subject to the Medical Research Involving Human Subjects Act (WMO), as confirmed by a non‐WMO declaration from the ethics committee. All participants provided informed consent prior to the interview.

**Results:**

Seven themes were generated from the data: feeling prepared for negative pressure wound therapy with instillation, gaining trust and hope because of wound healing, having trust in the expertise of nurses, facing fear and dread during negative pressure wound therapy with instillation, dealing with sleep disturbance, having difficulties with daily living activities, and worrying about undergoing negative pressure wound therapy with instillation at home. Although patients initially faced uncertainties, improved wound healing builds trust in the treatment.

**Conclusion:**

Negative pressure wound therapy with instillation significantly impacts patients' physical and emotional well‐being. Patients felt more prepared and confident when provided with detailed explanations about the device and what to expect during treatment. Nurses' unfamiliarity with the treatment further diminished patient trust. For optimal patient care, patients require adequate preparation and support from well‐trained nurses to manage the physical and emotional impact of negative pressure wound therapy with instillation, in both hospital and home settings.

**Reporting Method:**

Adheres to the Reflexive Thematic Analysis Reporting Guidelines.

**Patient or Public Contribution:**

Patients were involved in the study through interviews.

## 1 Introduction

During hospitalisation, patients may develop complex wounds due to various causes such as surgical complications, traumatic injuries, pressure ulcers, infections, or chronic diseases like diabetes mellitus. These wounds are often characterised by delayed healing, high exudate levels, necrosis, and recurrent infection. If not properly managed, they can lead to serious complications including local and systemic infections, sepsis, extended hospitalisation, and even mortality [1]. In Europe, the prevalence of complex wounds is approximately 3–4 per 1000 patients, representing a substantial clinical problem [2]. Complex wounds interfere with a patient's recovery by prolonging physical limitations and causing persistent pain and fatigue. These effects frequently extend beyond the physical, contributing to emotional stress, reduced independence, and social withdrawal. The ongoing need for intensive wound care and repeated interventions adds to the burden, often making it difficult for patients to maintain their quality of life. Quality of life is a multidimensional concept capturing physical, psychological, and social dimensions of an individual's well‐being [3, 4]. More specifically, well‐being refers not only to the absence of suffering, but to a dynamic balance shaped by interactions between the individual, their body, and the care environment, integrating physical, psychological, and social dimensions [4, 5].

Standard care for complex wounds involves a comprehensive approach that includes: debridement, infection control, moisture balance, pressure offloading, and management of comorbidities [1]. Despite these interventions, some complex wounds may not respond adequately, necessitating advanced therapeutic options [6]. One such innovation is negative pressure wound therapy with instillation (NPWTi). This therapy was introduced in 2012 as a new generation of negative pressure wound therapy (NPWT) [6, 7]. Traditional NPWT applies negative pressure through a foam dressing secured with a film and connected to an electric device that generates a vacuum [8]. A notable drawback of traditional NPWT is its limited ability to dissolve infected, devitalized, or necrotic tissue. NPWTi addresses this by instilling a topical solution onto the wound bed for a specified duration before removing it through negative pressure cycles [6, 7]. This method aims to enhance local blood flow, stimulate granulation tissue formation, reduce bacterial overgrowth and edema, and dilute and solubilise wound debris, making it especially suitable for wounds that are necrotic, heavily infected, or contain devitalized tissue that are less responsive to traditional NPWT [9, 10]. NPWTi has seen growing adoption and interest driven by its ability to combine wound cleansing with negative pressure therapy and by the increasing complexity of wounds requiring more advanced treatment strategies [6, 10].

Although NPWTi has predominantly been used in clinical settings, its application in home setting in the Netherlands is expected to rise in the near future, as traditional NPWT is widely implemented in this setting [11]. This transition raises important questions about how patients might experience continuing this therapy at home. They may face a shift in their care environment, which can influence their sense of safety, autonomy, and control over daily routines. Since patients may not have prior experience with NPWTi at home, understanding their expectations is crucial. Exploring these hypothetical expectations can help anticipate potential challenges, guide the development of patient‐centered support strategies, and ensure that home‐based NPWTi promotes both effective wound healing and well‐being [12].

The literature on NPWTi primarily focuses on clinical efficacy [13, 14]. A meta‐analysis suggests NPWTi may be superior to traditional NPWT, with fewer required surgeries, fewer dressing changes, and smaller wound areas [14]. Several studies highlight the significant impacts of traditional NPWT on patients' physical, psychological, and social well‐being [15, 16, 17, 18, 19]. A systematic review by Janssen et al. identified reduced mobility, decreased self‐esteem, increased dependency, and gained self‐control. Patients experienced pain and physical limitations from being attached to the device, distress from odours and noise, and limitations on work and social life [18]. These issues can negatively affect patient well‐being and wound healing [16]. A similar impact is expected with NPWTi, but additional factors affecting patients' physical, psychological, and social well‐being can be expected. NPWTi involves a larger device than NPWT and includes fluid instillation every 2–3 h, which often requires patients to lie down for 15 min. Patients' experiences with NPWTi during hospitalisation, and their expectations regarding continuing this treatment at home remain underexplored.

Understanding patient experiences and expectations is particularly important for nurses, who play a central role in delivering NPWTi, managing associated pain, ensuring hygiene, educating patients, providing mental support, and preparing them for discharge and home‐based care [18, 20]. Capturing these experiences and expectations can guide care improvements, enhance treatment adherence, and improve tailored support to reduce impact on patients' physical, psychological, and social well‐being.

This study aims to explore the physical and emotional experiences of hospitalised patients with all types of wounds undergoing NPWTi. The secondary aim is to explore patients' expectations regarding continuing treatment at home.

## 2 Methodology

### 2.1 Design

We conducted a qualitative study using semi‐structured interviews analysed through Reflexive Thematic Analysis (RTA), as described by Braun and Clarke [21]. This design enabled us to explore and interpret patients' experiences and expectations of NPWTi. We followed the RTA Reporting Guidelines (RTARG) to ensure transparency, reflexivity, and methodological rigour throughout the research process [21].

### 2.2 Study Setting and Participants

We recruited patients who were undergoing, or had recently received NPWTi treatment through a convenience‐based recruitment approach across eight hospitals in the Netherlands. This approach reflects both the small number of eligible patients and the ethical need to minimise burden for individuals in vulnerable health conditions. Recruitment aimed to be low‐threshold and accessible, enabling patients to share their experiences in a manner that respected their physical and emotional circumstances [22, 23]. Participants were identified through three routes: (1) surgical and gynecologic wards at a university medical center, (2) patients who had received NPWTi as part of an ongoing multicenter trial (trial registration: Overview of Medical Research in the Netherlands (OMON) ID NL‐OMON19878) and consented to be contacted for additional studies, and (3) distributing flyers via healthcare professionals at other hospitals.

Patients were eligible if they had received NPWTi for any type of wound for at least 3 days within the past year, were aged 18 years or older, were able to give informed consent, and communicate in Dutch. Exclusion criteria were cognitive deficiencies that might hinder engagement in an interview or if their life expectancy was less than 3 months, to avoid placing additional burden on patients in vulnerable circumstances.

Eligible patients were contacted by the first author (AN) either in person or by telephone to provide study information. Interested patients received an information letter, and informed consent was obtained verbally, by audio‐recording, or in writing, depending on the interview mode. Participants could choose between face‐to‐face or telephone interviews. We chose flexibility to enhance accessibility and participation for patients and to address a possible low inclusion rate [23]. The face‐to‐face interview took place in a private room at the hospital, and telephone interviews took place in a private room without other people present.

### 2.3 Data Generation

We conducted one‐time, semi‐structured interviews between January 2024 and June 2024. We developed the topic list provided in Table 1 based on clinical experience with NPWTi from members of the research team (HG and AME) and insights from previous studies on traditional NPWT, including decreased self‐esteem, anxiety, pain, reduced movement, and care quality [15, 16, 17, 18, 19, 24]. The topic list focused on exploring participants' physical, emotional, and social experiences with NPWTi and their hypothetical expectations about continuing treatment at home. Each interview started with the question: “What feeling comes to mind when I mention NPWTi?” During the data generation period, the topic list was reflexively refined to incorporate insights from previous interviews, allowing for greater depth and richness in subsequent interviews [23].

**TABLE 1:** Topiclist.

Topic 1: Physical experiences Topic 2: Mental experiences Topic 3: Going home with NPWTi Questions prior to the interview: What is your age? What is your primary diagnosis? How did the wound occur? Where is the wound located? What is your living situation? Do you have a caregiver? How long have you been treated with the pump?	
**Subjects**	**Topics**
Introduction and background	Introduce What the study is about Duration of the interview Mention audio recording of the conversation
Opening question	What comes to mind when we talk about the negative pressure wound therapy pump?
Topic 1: Physical experiences	Comfort Flush system (temperature, frequency, comfort, leakage) Dressing change (comfort, frequency) Suction (vacuum) Mobilisation Sleep Activities of daily living (ADL) Other experiences
Topic 2: Mental experiences	First time the pump was applied Emotional changes over time Alarms going off Anxiety Looking at and thinking about the wound Getting used to it Professional help Acceptance of the pump Dependency Visits from family and friends Other experiences
Topic 3: Going home with NPWTi	Expectations about going home Feeling apprehensive How long it will take to feel comfortable What is needed to go home safely and with confidence Home care staff Other experiences

All 12 interviews were conducted by the first author (AN), who was unknown to participants and had no clinical experience with NPWTi. One took place face‐to‐face in a private hospital room, while the remaining interviews were conducted by telephone, based on participant preference. The initial interview served as a pilot and was analysed prior to proceeding with further interviews to ensure the appropriateness and completeness of the topic list [23]. This pilot interview indicated that the topic guide was appropriate, requiring only minor adjustments for clarity and flow. Interviews lasted 21–42 min (mean: 32 min) and were audio‐recorded. After each interview, the first author (AN) wrote reflexive memos to capture contextual observations, participant behaviour, and immediate reflections [21]. An experienced qualitative researcher (HG) reviewed the first two recordings and provided feedback to enhance interview techniques to support ongoing reflexivity and rigour [23].

### 2.4 Data Analysis

We analysed the data using Braun and Clarke's six‐phase framework for Reflexive Thematic Analysis (RTA) [21]. We adopted an experiential and contextualist orientation, aiming to capture participants' lived experiences within the context of their care. The analysis was largely inductive, allowing us to generate themes from the data rather than being imposed by pre‐existing theory. The first author (AN) transcribed the interviews in detail, including pauses, to preserve the meaning and nuance, and engaged deeply with the data through repeated reading and note‐taking to develop familiarity and initial impressions. We coded the data inductively using MaxQDA software (version 2022) [25], focusing on segments of text that captured meaningful aspects of participants' experiences and expectations of NPWTi. We conducted the coding primarily at a semantic level, with latent interpretation applied when relevant to capture underlying meaning related to well‐being. To enhance reflexive depth, the last author (HG) independently read and coded the first two and seventh interviews. AN, HG and HW engaged in ongoing reflexive discussions, developing, reviewing and refining codes iteratively. Rather than seeking consensus or reliability, these focused on deepening reflexivity and interpretative insight. Following this, AN, HG and HW worked on combining codes and constructing potential themes that represented patterned meanings across the dataset. These themes were reviewed and refined in relation to the coded extracts and the overall dataset ensuring coherence, distinctiveness, and analytic richness. We then developed a thematic map visualising relationships among themes, with each theme illustrated using vivid, representative quotes. All quotations included in this manuscript were originally spoken in Dutch. The first author (AN) translated the quotations into English, carefully preserving the semantic meaning of participants' statements. During translation, attention was given to maintaining emotional tone, nuance, and context, ensuring that feelings such as frustration, relief, or anxiety were accurately conveyed. To enhance validity, selected translations were reviewed by a bilingual team member (HG) familiar with the study context. Consistent with RTA, we judged that the dataset held sufficient information power and meaning diversity to address the research aim after 12 interviews [21].

### 2.5 Ethical Considerations

We performed this study in line with the principles of the Declaration of Helsinki [26]. The Institutional Review Board of the tertiary hospital determined that ethical approval was not required under the Medical Research Involving Human Subjects Act (Reference 2023.0825) [27], as the study involved no interventions or study‐related procedures.

### 2.6 Reflexivity and Rigour

We ensured rigour through continuous reflexivity, transparency, and adherence to the RTARG [21]. We maintained reflexivity as an ongoing, critical awareness of our assumptions, positionality, and interpretive influence throughout the research process. Our team held regular discussions to reflect on how their professional backgrounds, disciplinary perspectives, and assumptions might influence data collection, analysis, and analytical focus. These conversations supported analytical decisions and encouraged ongoing reflection on developing interpretations and enhanced the trustworthiness of the analysis. We drew on our diverse expertise, encompassing clinical experience in wound care, qualitative research methodology, and personal experiences. This diversity was considered a resource, enriching the collection and analysis of the data through critical dialogue and ongoing reflexive consideration. We provided a detailed description of the research context, participant recruitment, and analytic procedures to enable readers to evaluate transferability and resonance of findings. We maintained reflexive memos throughout to document interpretative decisions. In line with the RTARG, we reported our methodological choices transparently, including recruitment approach, interview flexibility, transcription, and analytic strategy, thereby enhancing the study's credibility and coherence [21].

## 3 Results

Seventeen patients were recruited, with five declining due to personal circumstances, such as family‐related circumstances. Demographics are shown in Table 2. Five men and seven women participated, with a median age of 75.5 years (interquartile range 10.8). Two patients were recruited from the university hospital, eight through an ongoing trial, and two responded to the flyer. Patients received NPWTi at eight different hospitals across the Netherlands. Most patients had wounds caused by surgical site infections (SSI) following abdominal or vascular surgery, while one patient had an ischemic wound. Although most patients lived with a partner or family, only half of the patients could rely on them for care. The duration of NPWTi ranged from 3 days to over 2 weeks.

**TABLE 2:** Patient characteristics.

Characteristics	*n* (12)
Age (years) (median, IQR)	75.5 (10.8)
Gender (*n*)	
Male	5
Female	7
Cause of the wound (*n*)	
Vascular surgery	4
Abdominal surgery	6
Trauma surgery	1
Ischemia	1
Type of wound (*n*)	
Infected post‐operative wound	11
Ischemic wound	1
Wound site (*n*)	
Abdomen	6
Groin and hip	2
Lower extremities	4
Living situation (*n*)	
Alone	2
Not alone	10
Informal caregiver (*n*)	
Yes	6
No	6
Duration of NPWTi (*n*)	
3–6 days	3
7–10 days	3
11–14 days	3
> 14 days	3

Abbreviations: IQR, interquartile range; *n*, number.

**FIGURE 1:**
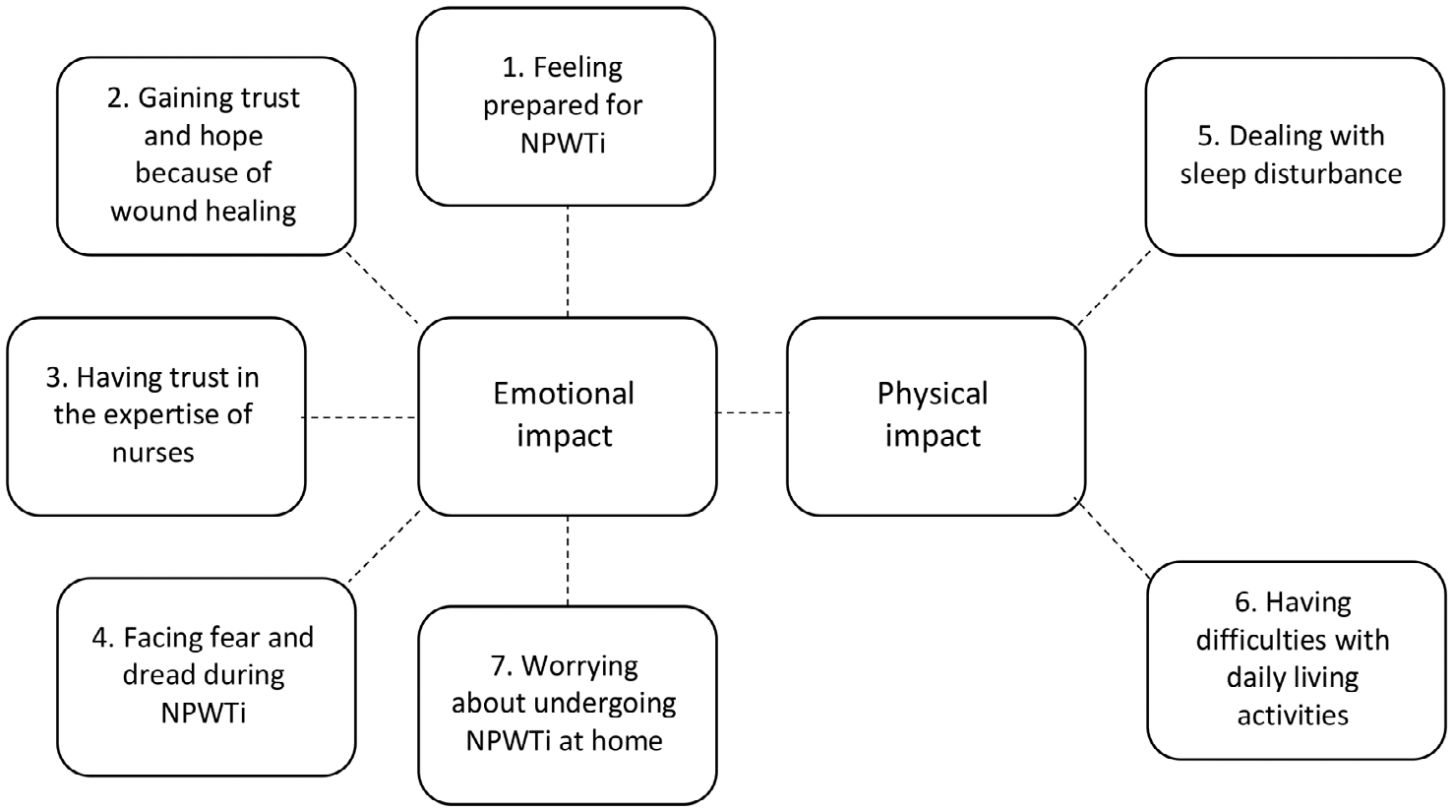
Thematic map with interconnections between themes.

We generated six themes for the physical, emotional and social well‐being experiences with NPWTi: (1) feeling prepared for NPWTi, (2) gaining trust and hope because of wound healing, (3) having trust in the expertise of nurses, (4) facing fear and dread during NPWTi, (5) dealing with sleep disturbance, and (6) having difficulties with daily living activities. For expectations at home, we generated one theme: (7) worrying about undergoing NPWTi at home. The themes were crafted into physical and emotional impacts and showed an interconnection between the themes, illustrated in a thematic map (Figure 1). Although social well‐being was explored in the interviews, patients' experiences related to social well‐being were often embedded within or overlapped with physical and emotional experiences. Therefore, we did not present social well‐being as a separate theme but recognise its influence across both physical and emotional impacts. According to RTA, we present the themes we generated through our analysis of the data, followed by a brief reflection in which we situate these themes within relevant existing research [21]. Table 3 provides the themes with illustrative participant quotations.

**TABLE 3:** Themes with illustrative participants' quotations.

Theme	Representative quotations
Feeling prepared for NPWTi	“Of course, I am interested in how such a device works, so I studied it a bit. The surgeon also explained it clearly, saying, ‘This will happen, and we would like you to do this and that.’ I was very well informed, and I could read the pump too. In three minutes, it would start rinsing again, so that was very helpful.” (P12) “I did not feel like I was told beforehand that I would have to lie down every two and a half hours for the pump to rinse. That was something that really disappointed me because I had to keep track of when those two and a half hours were over, so I was constantly focused on the pump.” (P5)
Gaining trust and hope because of wound healing	“I look at it positive now because with the dressing changes, we see small improvements every time. Initially, I did not think about it that way to be honest. Because I thought, here we go again, doing all sorts of things. Just remove the infected part of the stoma, and I hope I can continue with my life. But now it is giving positive results. So, now I am positive about it myself.” (P7)
Having trust in the expertise of nurses	“They did not really know much about the pump. Some of them just did not know how to use it or what to do when it malfunctioned. Some did not even know how to turn it off and still had to ask each other how to do it. I think it is normal that they have some idea of how it works. So, that caused stress sometimes, because, yes, you just want things to go well and not have your stomach wound suffer… I kept a very close eye on them and pointed out when they forgot something.” (P11) “Yes, the guidance was really good. There was a nurse who gave the course on how to set up the pump and she was always there with me. So, when the nurses had to do it, since they all had to learn it, she was there to explain and supervise it. It went really well, and I think I was lucky that she was there all the time.” (P2)
Facing fear and dread during NPWTi	“Well, applying the foam was not a pleasant feeling, because the wound, it was about four centimetres deep into the abdominal cavity, so all that stuff had to be put in there, and that is not fun at all… So yes, that is the only big thing where you think, tomorrow that foam needs to be replaced again.” (P3) “The first time I saw it (the wound), when it was opened, it was a very deep hole. A very big one, and although it is my own, it is quite an unpleasant sight.” (P2)
	“The leakages happened more often and that was quite painful with the changing. They would pull out the bandage and they would pull out the gauze, and then they had to put in new gauze again. There were days when it was once a day, and there were days when it was twice, but I also had days when it was more. One time it even happened at night and then it all had to be cleaned completely. That was not very pleasant, not pleasant at all, because it did not want to look at the wound and feel the pain again.” (P4)
Dealing with sleep disturbance	“Yeah, that is a bummer of course. I already do not sleep well in the hospital, and when you finally doze off and that thing starts beeping, well, that is not pleasant. Sometimes they had to take off all the foam and repack it. They would be busy with that for like half an hour, sometimes up to three‐quarters of an hour.” (P3) “When it alarmed and they could not fix it, it kept going off. We once had a pump that alarmed every 5 to 10 min, and they just could not stop it. So, I did not sleep at all that night, because of the pump. That was very disturbing, and I felt very tired after that night.” (P11)
Having difficulties with daily living activities	“The wound hurts when I walk and you have to be really careful that you bring all the wires and tubes, because you would not want to get stuck behind something. So in the beginning I was demotivated to go for a walk because I did not feel like having to deal with everything.” (P7) “That was something that really bothered me, because I had to keep track of when the two and a half hours were up, so I was constantly focused on that pump… And some sort of ring was placed around it, to make it flow in more easily, but I had to lie on my side for that. And I could not do it alone, so I had to keep calling the nurses. I think, if there was a signal, like ‘in ten minutes you need to lie down’, but that did not happen either, so I had to keep setting my alarm and that made me really uncertain.” (P5)
Worrying about undergoing NPWTi at home	“I think I would have found that difficult, because… you know, those obstacles continue at home and now that I do not have the pump anymore, I feel a lot freer.” (P5) “My whole life will be completely different… I will have to keep in mind that people will come to my house and if I want to get up I need to take the pump with me. I do not think that is what a human wants. At least I do not.” (P1)

### 3.1 Feeling Prepared for NPWTi

Patients shared that preparation and information provision played a key role in shaping their sense of confidence and control during NPWTi treatment. Several patients described receiving detailed explanations about the mechanism of the device and what to expect during treatment. Patients expressed that this comprehensive information helped them feel more prepared and confident in their therapy. They received information verbally by the wound nurse or doctor and were encouraged to ask questions if anything was unclear. Patients included through the ongoing trial received additional information via a comprehensive patient information letter of the trial, which contained details on the working mechanism of the device and potential discomforts.

However, not all patients felt adequately prepared. They described discovering key aspects of the treatment only after initiation, such as the frequency of instillation cycles or required positioning, which led to unexpected challenges and confusion during treatment.

These contrasting experiences illustrate how clarity and completeness of pre‐treatment communication directly influenced patients' emotional adjustment to NPWTi. This aligns with Jon Links et al. [28], who emphasise that effective communication, characterised by timely information, participatory dialogue, and empowerment, supports patients' confidence, engagement, and perceived safety in care. In the context of NPWTi, structured and accessible information provision can foster both practical preparedness and psychological comfort, helping patients to feel more in control of their treatment and daily routines.

### 3.2 Gaining Trust and Hope Because of Wound Healing

During NPWTi, patients' initial concerns regarding efficacy shifted to optimism as they observed wound size reduction and received encouraging feedback from healthcare professionals. Patients described that seeing debris removed through the tubes reassured them of thorough cleaning of the wound, fostering feelings of relief and hope regarding wound closure. They mentioned that this progress motivated them to continue treatment and trust its effectiveness.

These experiences show that observing wound improvements contributes to patients' emotional well‐being and confidence in their care. This aligns with findings from qualitative studies on traditional NPWT, where patients initially experienced anxiety and uncertainty but later found hope through wound healing [29].

### 3.3 Having Trust in the Expertise of Nurses

At the onset of NPWTi, patients expressed anxiety and lacked confidence in its efficacy, especially since they were among the first to undergo this treatment in their hospital. They felt more uncertain when nurses seemed unfamiliar with installing the device correctly or handling alarms. Patients mentioned that sometimes nurses spent up to 2 h changing dressings, frequently seeking assistance, which led to concerns about the impact on their wounds, prompting them to pay close attention.

Some patients felt reassured when nurses explained their actions and showed effort to ensure the best care. Additionally, patients found comfort knowing nurses had received specialised training or were supervised by experienced nurses during NPWTi administration.

These experiences show that patients' perceptions of nurses' competence actively shaped their trust, emotional comfort, and engagement with NPWTi. This is supported by a systematic review highlighting that patients' trust is strongly influenced by nurses' knowledge, technical skill, and confidence in care delivery [30].

### 3.4 Facing Fear and Dread During NPWTi

Patients described experiencing discomfort during different phases of NPWTi. They often indicated that dressing changes, typically required every 2–3 days and involving removing old foam and inserting new, were painful. Patients with deep wounds reported that their discomfort was further intensified because the foam had to be packed deeper into the wound. They felt anxious, particularly the day before and on the day of the dressing change. In certain cases, the dressing change could last up to 2 h, prolonging their discomfort. To manage pain, patients mentioned that they received numbing drops or underwent dressing changes under anaesthesia in the operating room. Patients expressed discomfort when seeing the size and depth of their wounds, particularly during initial dressing changes, finding it unpleasant to confront.

Patients described that the fluid instillation every 2–3 h was painful, intensifying their dread of this phase. Patients indicated that leakage through the dressing sometimes occurred, causing device alarms and dampened beds, necessitating new dressing changes, which amplified their fear and dread.

These experiences illustrate that both physical pain and emotional distress are intertwined during NPWTI, emphasising the importance of effective pain management, clear guidance, and psychological support. This aligns with prior research on traditional NPWT, which highlights that pain and fear during wound care can negatively affect patient well‐being and engagement with treatment [16].

### 3.5 Dealing With Sleep Disturbance

Patients described sleep disturbances caused by the NPWTi device, attributing this to periodic fluid instillation every 2–3 h, continuous buzzing sounds, or alarms. They mentioned that pain from fluid instillation woke them, making it difficult to fall back asleep. Leakage often necessitated immediate dressings changes during the night. Alarms were also triggered by empty fluid bags, largely due to scheduling issues among nursing staff. Consequently, patients experienced daytime fatigue and lacked the energy to engage in daily activities. To compensate, they mentioned napping during the day and expressed frustration when nurses checked their vitals during these naps, further reducing their desire for outdoor activities or family visits.

These experiences indicate that NPWTi can significantly compromise sleep quality, affecting both physical and psychological recovery. Adequate sleep is essential for immune function, which plays a vital role in wound healing [31]. Patients who are preoccupied with their treatment and experience disrupted rest are at higher risk of reduced mental well‐being, which can negatively affect wound healing [32].

### 3.6 Having Difficulty With Daily Living Activities

Patients experienced mobility difficulties due to the presence of tubes, the size of the device, wound pain, fatigue, and the inconvenience of carrying the device. This discouraged them from engaging in walking activities. Additionally, they felt uncomfortable being observed when walking, sensing judgement from others about their condition. While patients managed to perform basic hygiene tasks and use the toilet, either with or without assistance from nurses, most refrained from showering with the device due to concerns about water damage or discomfort from the tubes.

Patients with wounds on their abdomen or hip mentioned that they often had to lie flat during fluid instillation to ensure proper treatment. This required them to constantly monitor the time and set alarms to maintain the instillation schedule. Despite their continuous alertness, some patients missed timings, causing anxiety and restricting their mobility. Patients suggested having the device signal them before and after each instillation to regain a sense of control. In contrast, bedridden patients felt not required to constantly monitor the clock, allowing them to experience a lower level of anxiety.

These experiences demonstrate that NPWTi can significantly disrupt daily routines, contributing to feelings of dependency, stress, and reduced autonomy. Earlier research indicates that mobility and self‐care are crucial for minimising physical complications and enhancing well‐being; hence, interventions that support patient mobility and facilitate daily activities during NPWTi are essential [33].

### 3.7 Worrying About Undergoing NPWTi at Home

Patients expressed concerns about the potential continuation of NPWTi at home. They were worried about the significant responsibilities involved and emphasised the importance of receiving proper training from experienced nurses to understand and manage the device. Patients with informal caregivers at home felt more supported, reducing their concerns. Furthermore, patients worried about the ongoing discomfort at home and underscored the importance of knowing whom to contact in case of issues, as they would no longer have nurses as easily accessible at home as they did in the hospital.

Patients talked about their willingness to have home care professionals visit to assist with dressing changes. They mentioned being comfortable with home care visits and would just schedule their day around the appointments. However, others expressed reservations about scheduling around the visiting times and the inconvenience of needing assistance, especially during nighttime hours. Patients mentioned concerns about the pump alarming at night and the need to call someone to come by for support.

These hypothetical expectations reflect patients' concerns regarding self‐management, continuity of care, and potential limitations in daily living. Consistent with earlier research, inadequate instruction and preparation can generate unnecessary stress when patients manage NPWT at home [34].

## 4 General Discussion

This study explored patients' experiences with NPWTi and their hypothetical expectations regarding its potential of continuing treatment at home. Seven interconnected themes, categorised into physical and emotional impacts, were revealed, with social well‐being intertwined with or overlapping both. Emotionally, patients felt more prepared and confident when provided with detailed explanations about the device and what to expect during treatment. Observing positive progress in wound healing increased their trust in the treatment. However, nurses' unfamiliarity with NPWTi raised concerns about its potential impact on their wounds. Furthermore, patients expressed anxiety about dressing changes, fluid instillation, and seeing their wound. Concerns about going home with NPWTi were caused by the responsibilities involved and the need for home care visits. Physically, frequent alarms, buzzing sounds, and fluid instillation disrupted sleep, causing daytime fatigue, reduced mobility, and limited social interactions. Daily activities were further affected by challenges of managing tubes, wound pain, the size of the device and the inconvenience of carrying it. Patients who had to lie down during fluid instillation felt anxious about adherence to the instillation schedule. These experiences reveal that NPWTi affects patients in a multifaceted way, with physical, emotional and social experiences closely interrelated and shaping overall well‐being.

As patients observed improvements in wound healing because of NPWTi, their trust and hope increased. This aligns with findings from a qualitative study on traditional NPWT, where patients initially struggled with the device but later found hope through wound healing [29]. However, trust and hope are not only outcomes of physical improvement. The Unitary Caring Science Resilience‐Building (UCSRB) model emphasises that these qualities are deeply rooted in relational care, arising from meaningful interactions between patients and healthcare professionals [35]. In line with this model, patients in our study reported feeling anxious when nurses appeared unfamiliar with the device, underscoring the importance of well‐trained and competent nursing staff. This is supported by a systematic review demonstrating that patient trust is closely linked to nurses' knowledge and competence [30]. Furthermore, previous research has identified variability in the delivery and content of postoperative wound care education, highlighting the need for strategies to improve its consistency and comprehensiveness [36]. According to the UCSRB model, emotional well‐being, hope, and trust develop through relational interactions in which nurses validate patient fears and support autonomy, thereby reinforcing engagement with care [35]. Thus, while the trust and hope fostered by wound healing are beneficial, there is an urgent need for adequately trained nurses and effective educational materials to ensure patients are well‐prepared, reduce patients' fears and concerns, and ultimately improve both wound healing and the overall patient experience.

Patients frequently experienced a burden from dressing changes and the instillation of fluid every 2–3 h. The pain and visibility of the wound made dressing changes particularly distressing, consistent with findings on traditional NPWT [16]. Compared to conventional wound care, NPWTi typically involves less frequent dressing changes, usually every 2–3 days, thus reducing the frequency of painful interventions. However, its use in often complex and frequently infected wounds tends to result in heightened pain and discomfort during dressing changes [9, 10]. The instillation of fluid occasionally caused additional pain, increasing patients' distress and significantly impacting their well‐being [37]. This underscores the importance of effective pain management [32, 33]. Pain management can be addressed through a combination of strategies, including pharmacological options, such as pain medication, as well as non‐pharmacological approaches. These may include using relaxation techniques and providing clear explanations to patients about the procedure [38, 39]. Educating patients about the process can help alleviate fear and reduce perceived pain, as understanding the treatment reduces uncertainty and anxiety [40]. Despite these challenges, many patients reported that witnessing the rapid progress of their wound healing with NPWTi provided hope, motivating them to endure the discomfort.

Previous studies on traditional NPWT have identified physical limitations due to the attached device [15, 16, 17, 18, 19]. In the current study, patients faced even greater challenges, as NPWTi involves a larger device, periodic fluid instillation, and the treatment of more complex wounds. Many patients struggled with daily activities due to the difficulty of navigating around tubes. Those required to remain horizontal during instillation faced additional burdens, staying alert and preoccupied with adhering to their instillation schedule. Additionally, feelings of self‐consciousness, driven by a perceived judgement by others while walking with the device, further demotivated patients from staying active. These limitations are concerning because mobility and self‐care are crucial for minimising physical complications and enhancing overall well‐being [33, 41]. The impact on daily activities was further compounded when patients' sleep was disrupted by alarms, continuous buzzing sounds, and the need for fluid instillation—sometimes requiring a mandatory position—every 2–3 h. Frequent nighttime disturbances resulted in daytime fatigue, leading to increased daytime sleep and reduced activity. Quality sleep is crucial for immune function, which plays a vital role in wound healing [31]. Furthermore, patients preoccupied with their treatment are more likely to experience lower mental well‐being, negatively affecting wound healing [32]. Nurses must be aware of the effects NPWTi has on mobility, sleep and self‐care, and take steps to reduce patients' discomfort. This can be achieved by identifying and discussing these concerns with patients, addressing their needs and preferences to reduce sleep disturbance, and improve self‐management and self‐care. Nurses should clearly explain the instillation schedule, assist patients in managing it, and encourage them to plan and engage in daily activities. Additionally, nurses can manage the instillation schedule during the night, including setting alarms, so that patients can sleep uninterrupted and avoid the need to wake up for fluid instillation. By addressing mobility, sleep, and self‐care concerns in a caring and supportive manner, nurses reinforce patients' trust, hope, and engagement with treatment, while promoting both physical recovery and emotional resilience [35].

In the Netherlands, the use of NPWTi in home setting is expected to increase in the near future, making it essential to assess patients' expectations and tailor their support accordingly. Our study highlights the challenges patients face regarding the continuation of NPWTi at home. Although our sample may not fully represent the future population likely to be discharged with NPWTi, due to median age and the absence of an informal caregiver in half of the participants, the findings underscore the critical need for comprehensive support and education for both patients and caregivers. Patients expressed concerns about the challenges and responsibilities of managing NPWTi at home, including sleep disturbances, mobility and self‐care difficulties, and feelings of fear during treatment procedures. Additionally, their trust was diminished due to encounters with inexperienced nurses, leading to concerns about the need for professional home care. A particular concern was the lack of hospital‐level support at home. NPWTi at home may be most suitable for patients who are mobile, have an informal caregiver, can navigate with the device in their home environment, and are trained to handle basic tasks and alarms. A qualitative study on patients' experiences with traditional NPWT at home reported unnecessary stress due to insufficient instructions on how to use the device [34]. Another study on health literacy highlights the importance of accessible, patient co‐designed education materials [42]. Again, these findings reinforce the importance of thorough preparation and patient‐centered education. Nurses must ensure that both patients and informal caregivers are well‐instructed in managing NPWTi at home. In addition, district nurses should receive extensive NPWTi training, be easily accessible for ongoing patient support, and understand the impact of the treatment. Such comprehensive preparation and support can significantly reduce stress, enhance patients' confidence, and improve their overall well‐being [17, 18, 33].

## 5 Strengths and Limitations

The inclusion of patients with diverse wound sites and treatment durations across multiple hospitals in the Netherlands enabled the exploration of diverse patient experiences and contextual factors influencing NPWTi treatment. The first author (AN) prepared thoroughly for the interviews by reviewing relevant literature, attending a conference on NPWTi, and analysing several interviews in collaboration with an experienced NPWTi researcher (HG). Recruiting patients was challenging, as the number of patients with NPWTi at the university hospital was low. Therefore, patients were also recruited from an ongoing clinical trial and passive sampling with flyers. These approaches may have attracted participants who were particularly motivated to share their experiences, potentially shaping the nature and richness of the narratives generated. Most participants had surgical site infections, a common indication for NPWTi [43]. Although this may limit transferability to other wound types, the experiences primarily reflect the physical, emotional, and social impact of NPWTi and therefore still support the broader aim of the study [44]. 11 of the 12 interviews were conducted by telephone. Telephone interviews can effectively collect rich and detailed narratives, even on sensitive topics, by offering privacy and convenience for participants. This approach likely enabled participation from patients who might otherwise have been unable or unwilling to engage in face‐to‐face interviews, while still capturing detailed accounts of their experiences with NPWTi. However, as non‐verbal cues were less accessible, the first author (AN) paid closer attention to tone, pauses, and hesitation, and used follow‐up questions to clarify emotions and experiences that might otherwise have been conveyed non‐verbally [45, 46].

## 6 Implications and Future Research

This study reveals the significant impact NPWTi can have on patients' physical, emotional, and social well‐being. Nurses' unfamiliarity with the treatment can negatively affect patient trust, making it crucial to have well‐trained nurses supported by ongoing education. Well‐trained nurses ensure better wound care, provide effective support for patients during NPWTi, address patients' concerns, reduce the physical, emotional, and social impact, and ultimately improve patient satisfaction. To ensure the safe and effective use of NPWTi in the home setting, patients and informal caregivers must receive thorough, accessible, and patient‐centered education. Our findings regarding patient expectations for home‐based NPWTi provide valuable insights for developing comprehensive preparation and support strategies. These measures are essential to reduce stress, build patient confidence, and improve well‐being. Given the promising clinical outcomes of NPWTi, its use is expected to increase in the future, making these insights of great importance in providing the best care [13, 14].

Future research should focus on identifying strategies to improve patient satisfaction with NPWTi, such as enhancing nurse education. Additionally, there is a need for the development of consistent and comprehensive patient education that ensures patients are well‐prepared and helps reduce their fears and concerns. By addressing these areas, care for NPWTi patients can be optimised, and both treatment outcomes and overall patient satisfaction can be improved.

## 7 Conclusion

While patients initially experienced uncertainties with NPWTi, the positive outcomes in wound healing helped foster trust in the treatment. However, several physical, emotional, and social well‐being challenges developed, including disrupted sleep, mobility limitations, fear, and concerns about possible treatment continuation at home. Additionally, nurses' unfamiliarity with NPWTi further diminished patient trust. It is crucial for nurses to recognise these challenges and understand their substantial impact on patients' well‐being. Comprehensive nurse training is essential to optimally prepare and support patients and to deliver adequate wound care throughout the NPWTi treatment process, both in the hospital and at home.

## Author Contributions

A.N., H.G. and A.M.E. made substantial contributions to the research protocol and sampling strategies. A.N. was responsible for data collection and analysis, with H.G. involved in reviewing the quality of data collection and contributing to the analysis review. H.W. provided support in revising the data analysis for important intellectual content. A.N. and H.G. were responsible for drafting the manuscript. H.W., R.G.O., M.A.B., A.M.E. and H.G. critically revised the manuscript. All authors gave final approval of the version to be published. All authors have participated sufficiently in the work to take public responsibility for relevant portions of the content. The authors declare that generative artificial intelligence (AI) tools were used to improve the writing style of this manuscript

## Funding

The authors have nothing to report.

## Disclosure

The information reported in the manuscript has not been presented previously. There are no other papers that use the same dataset. Due to ethical concerns, the data supporting this study's findings are not publicly available.

## Ethics Statement

The Institutional Review Board of the tertiary hospital decided that ethical approval was not required for this study, as defined by the Medical Research Involving Human Subjects Act (Reference 2023.0825), as participants were not subjected to interventions or study‐related procedures. Depending on the mode of the interview, informed consent was obtained either verbally, through a recording, or in writing.

## Conflicts of Interest

M.A.B. reported receiving institutional grants from J&J and Solventum, and being a speaker and/or instructor for J&J, Solventum/3M, BD, Gore, Smith & Nephew, TelaBio, Angiodynamics, GDM, Medtronic, and Molnlycke outside the submitted work. A.M.E. received a European Wound Management grant outside the submitted work. M.A.B. and A.M.E. received a grant from ZonMw to investigate the effectiveness of NPWTi in a randomized clinical trial.  Other than the use of AI tools to improve the writing style, no other disclosures were reported.

## Data Availability

The data that support the findings of this study are available from the corresponding author upon reasonable request.

## References

[1] A. Labib and R. Winters , Complex Wound Management (StatPearls, 2025). 35015410

[2] R. Marques , M. Lopes , P. Ramos , J. Neves‐Amado , and P. Alves , “Prognostic Factors for Delayed Healing of Complex Wounds in Adults: A Scoping Review,” International Wound Journal 20, no. 7 (2023): 2869–2886. 36916415 10.1111/iwj.14128 PMC10410354

[3] C. R. Ratliff and V. Rovnyak , “Impact of Complex Wounds on Health‐Related Quality of Life,” Journal of Wound, Ostomy, and Continence Nursing 48, no. 6 (2021): 504–509. 10.1097/WON.0000000000000824 34781305

[4] D. Teoli and A. Bhardwaj , Quality of Life (StatPearls, 2025). 30725647

[5] G. Simons and D. Baldwin , “A Critical Review of the Definition of “Wellbeing” for Doctors and Their Patients in a Post Covid‐19 Era,” International Journal of Social Psychiatry 67, no. 8 (2021): 984–991. 34240644 10.1177/00207640211032259 PMC8592098

[6] P. J. Kim , C. E. Attinger , T. Constantine , et al., “Negative Pressure Wound Therapy With Instillation: International Consensus Guidelines Update,” International Wound Journal 17, no. 1 (2020): 174–186. 31667978 10.1111/iwj.13254 PMC7003930

[7] C. Lessing , P. Slack , K. Z. Hong , D. Kilpadi , and A. McNulty , “Negative Pressure Wound Therapy With Controlled Saline Instillation (NPWTi): Dressing Properties and Granulation Response In Vivo,” Wounds 23, no. 10 (2011): 309–319. 25881108

[8] J. Apelqvist , C. Willy , A. M. Fagerdahl , et al., “EWMA Document: Negative Pressure Wound Therapy,” Journal of Wound Care 26, no. Sup3 (2017): S1–S154. 10.12968/jowc.2017.26.Sup3.S1 28345371

[9] R. E. Horch , C. Braumann , J. Dissemond , et al., “Use of Negative Pressure Wound Therapy With Instillation and Dwell Time for Wound Treatment ‐ Results of an Expert Consensus Conference,” Zentralblatt für Chirurgie 143, no. 6 (2018): 609–616. 30537781 10.1055/a-0713-0517

[10] L. De Pellegrin , P. Feltri , G. Filardo , et al., “Effects of Negative Pressure Wound Therapy With Instillation and Dwell Time (NPWTi‐d) Versus NPWT or Standard of Care in Orthoplastic Surgery: A Systematic Review and Meta‐Analysis,” International Wound Journal 20, no. 6 (2023): 2402–2413. 36594491 10.1111/iwj.14072 PMC10333051

[11] Y. Huang , B. Mao , J. Hu , et al., “Consensus on the Health Education of Home‐Based Negative Pressure Wound Therapy for Patients With Chronic Wounds: A Modified Delphi Study,” Burns & Trauma 9 (2021): tkab046. 34993255 10.1093/burnst/tkab046 PMC8717889

[12] C. Eskilsson and G. Carlsson , “Feeling Confident in Burdensome Yet Enriching Care: Community Nurses Describe the Care of Patients With Hard‐To‐Heal Wounds,” QHW 5, no. 3 (2010): 5415. 10.3402/qhw.v5i3.5415 PMC2958087 20967140

[13] A. S. Timmer , P. R. Zwanenburg , A. M. Eskes , R. Hompes , and M. A. Boermeester , “The Effect of Negative‐Pressure Wound Therapy With Instillation Compared to Current Standard Care on Wound Closure Time of Infected Wounds: A Systematic Review and Meta‐Analysis,” Plastic and Reconstructive Surgery 150, no. 1 (2022): 176e–188e. 10.1097/PRS.0000000000009232 35583955

[14] G. Wang , H. Xu , G. Xu , H. Zhang , Z. Li , and D. Liu , “Clinical Outcomes of Negative Pressure Wound Therapy With Instillation vs Standard Negative Pressure Wound Therapy for Wounds: A Meta‐Analysis of Randomised Controlled Trials,” International Wound Journal 20 (2023): 1739–1749. 36519410 10.1111/iwj.13989 PMC10088847

[15] C. B. Thorup , M. Hougaard , P. F. Blindum , and E. E. Sørensen , “Hospitalised Patients' Experiences During Negative Pressure Wound Therapy due to Surgical Site Infection After Vascular and Cardiac Surgery,” International Wound Journal 15, no. 5 (2018): 707–716. 29927043 10.1111/iwj.12913 PMC7950218

[16] D. Upton , D. Stephens , and A. Andrews , “Patients' Experiences of Negative Pressure Wound Therapy for the Treatment of Wounds: A Review,” Journal of Wound Care 22 (2013): 34–39. 23299356 10.12968/jowc.2013.22.1.34

[17] A. H. J. Janssen , E. H. H. Mommers , J. Notter , T. S. de Vries Reilingh , and J. A. Wegdam , “Negative Pressure Wound Therapy Versus Standard Wound Care on Quality of Life: A Systematic Review,” Journal of Wound Care 25, no. 3 (2016): 154–159. 26947696 10.12968/jowc.2016.25.3.154

[18] A. H. Janssen , J. A. Wegdam , T. S. de Vries Reilingh , A. M. Eskes , and H. Vermeulen , “Negative Pressure Wound Therapy for Patients With Hard‐To‐Heal Wounds: A Systematic Review,” Journal of Wound Care 29, no. 4 (2020): 206–212. 32281512 10.12968/jowc.2020.29.4.206

[19] A. Miyanaga , T. Miyanaga , K. Sakai , C. Konya , K. Asano , and K. Shimada , “Patient Experience of Negative Pressure Wound Therapy: A Qualitative Study,” Nursing Open 10, no. 3 (2023): 1415–1425. 36199166 10.1002/nop2.1392 PMC9912402

[20] N. A. Tayyib and P. Ramaiah , “Nurses' Challenges in Wound Care Management‐A Qualitative Study,” Journal of Clinical and Diagnostic Research 15 (2021): 14626.

[21] V. Braun and V. Clarke , “Supporting Best Practice in Reflexive Thematic Analysis Reporting in Palliative Medicine: A Review of Published Research and Introduction to the Reflexive Thematic Analysis Reporting Guidelines (RTARG),” Palliative Medicine 38, no. 6 (2024): 608–616. 38469804 10.1177/02692163241234800 PMC11157981

[22] I. Etikan , “Comparison of Convenience Sampling and Purposive Sampling,” American Journal of Theoretical and Applied Statistics 5, no. 1 (2016): 1.

[23] U. Flick , An Introduction to Qualitative Research, 7th ed. (SAGE Publications, 2022).

[24] Y. Huang , J. Hu , B. Mao , et al., “Perspectives on the Process of Negative Pressure Wound Therapy at Home in Patients With Chronic Wound: A Qualitative Descriptive Study,” International Journal of Lower Extremity Wounds 21, no. 4 (2022): 384–396. 32772902 10.1177/1534734620946577

[25] VERBI Software , “MAXQDA 2022,” (2021), https://www.maxqda.com/.

[26] The World Medical Association INC , “Declaration of Helsinki Ethical Principles for Medical Research Involving Human Subjects,” (2008).

[27] Medical Research Involving Human Subjects Act (WMO) , “Central Committee on Research Involving Human Subjects,” (2023).

[28] M. Links , L. Watterson , P. Martin , S. O'Regan , and E. Molloy , “Finding Common Ground: Meta‐Synthesis of Communication Frameworks Found in Patient Communication, Supervision and Simulation Literature,” BMC Medical Education 20, no. 1 (2020): 45. 32046704 10.1186/s12909-019-1922-2 PMC7014645

[29] J. Abbotts , “Patients' Views on Topical Negative Pressure: ‘Effective but Smelly,” British Journal of Community Nursing 19, no. Sup10 (2010): S37–S41. 10.12968/bjon.2010.19.Sup10.79692 21072010

[30] K. Rørtveit , B. Sætre Hansen , I. Leiknes , I. Joa , I. Testad , and E. Severinsson , “Patients' Experiences of Trust in the Patient‐Nurse Relationship—A Systematic Review of Qualitative Studies,” Open Journal of Nursing 05, no. 3 (2015): 195–209.

[31] J. Balikji , M. M. Hoogbergen , J. Garssen , T. Roth , and J. C. Verster , “Insomnia Complaints and Perceived Immune Fitness in Young Adults With and Without Self‐Reported Impaired Wound Healing,” Medicine 58, no. 8 (2022): 1049. 10.3390/medicina58081049 PMC9412748 36013516

[32] S. Basu , A. G. Goswami , L. E. David , and E. Mudge , “Psychological Stress on Wound Healing: A Silent Player in a Complex Background,” International Journal of Lower Extremity Wounds 23 (2022): 365–371. 35102769 10.1177/15347346221077571

[33] G. Hussey and T. Young , “The Importance of Patient Wellbeing,” Wounds International 11, no. 4 (2020): 58–62.

[34] C. Monsen , S. Acosta , and C. Kumlien , “Patients Experiences of Negative Pressure Wound Therapy at Home for the Treatment of Deep Perivascular Groin Infection After Vascular Surgery,” Journal of Clinical Nursing 26, no. 9–10 (2017): 1405–1413. 28001332 10.1111/jocn.13702

[35] H. Wei , S. Hardin , and J. Watson , “A Unitary Caring Science Resilience‐Building Model: Unifying the Human Caring Theory and Research‐Informed Psychology and Neuroscience Evidence,” International Journal of Nursing Sciences 8, no. 1 (2021): 130–135. 33575453 10.1016/j.ijnss.2020.11.003 PMC7859535

[36] B. M. Gillespie , R. Walker , F. Lin , et al., “Nurse‐Delivered Patient Education on Postoperative Wound Care: A Prospective Study,” Journal of Wound Care 32, no. 7 (2023): 437–444. 37405945 10.12968/jowc.2023.32.7.437

[37] D. Upton and A. Andrews , “Pain and Trauma in Negative Pressure Wound Therapy: A Review,” International Wound Journal 12, no. 1 (2015): 100–105. 23489350 10.1111/iwj.12059 PMC7950877

[38] Y. Ma , Y. Li , C. Wang , et al., “Effects of Non‐Pharmacological Interventions on Pain in Wound Patients During Dressing Change: A Systematic Review,” Nursing Open 11, no. 2 (2024): e2107. 38391098 10.1002/nop2.2107 PMC10830920

[39] A. Purcell , T. Buckley , J. King , W. Moyle , and A. P. Marshall , “Topical Analgesic and Local Anesthetic Agents for Pain Associated With Chronic Leg Ulcers: A Systematic Review,” Advances in Skin & Wound Care 33, no. 5 (2020): 240–251. 32304447 10.1097/01.ASW.0000658572.14692.fb

[40] L. F. Callender , A. L. Johnson , and R. M. Pignataro , “Patient‐Centered Education in Wound Management: Improving Outcomes and Adherence,” Advances in Skin & Wound Care 34, no. 8 (2021): 403–410. 34260418 10.1097/01.ASW.0000753256.29578.6c

[41] M. J. Javed and D. D. Davis , Assisting Patients With Mobility (StatPearls Publishing LLC, 2024). 32644526

[42] I. O. Falade , J. C. Wilson , M. E. Mehari , D. Soroudi , S. Song , and E. A. Kim , “The Complexity of Online Patient Education Materials for Wound Care Strategies: A Readability Analysis,” Surgery 176, no. 2 (2024): 324–330. 38769036 10.1016/j.surg.2024.04.014

[43] J. Acosta , L. Galarza , M. Marsh , R. Martinez , M. Eells , and A. Collinsworth , “Effectiveness of Negative Pressure Wound Therapy With Instillation and Dwell in Removing Nonviable Tissue, Promoting Granulation Tissue, and Reducing Surgical Debridements: A Systematic Literature Review,” Wound Repair and Regeneration 33, no. 4 (2025): e70059. 40583835 10.1111/wrr.70059 PMC12207569

[44] V. Braun and V. Clarke , Thematic Analysis: A Practical Guide (SAGE Publications, 2021).

[45] L. Drabble , K. Trocki , B. Salcedo , P. Walker , and R. Korcha , “Conducting Qualitative Interviews by Telephone: Lessons Learned From a Study of Alcohol Use Among Sexual Minority and Heterosexual Women,” Qualitative Social Work 15, no. 1 (2016): 118–133. 26811696 10.1177/1473325015585613 PMC4722874

[46] M. Saarijärvi and E. Bratt , “When Face‐To‐Face Interviews Are Not Possible: Tips and Tricks for Video, Telephone, Online Chat, and Email Interviews in Qualitative Research,” European Journal of Cardiovascular Nursing 20, no. 4 (2021): 392–396. 33893797 10.1093/eurjcn/zvab038 PMC8135391

